# Missed opportunities: delayed in-hospital palliative care consultations in patients with non-metastatic cancers

**DOI:** 10.1007/s00520-026-11196-7

**Published:** 2026-09-09

**Authors:** Christopher Paschen, Klemens Pinter, Thomas Martin Hofbauer, Irene Tschischka, Claudia Wenzel, Feroniki Adamidis, Oliver Robak, Eva Katharina Masel

**Affiliations:** 1 Division of Nephrology and Dialysis, Department of Medicine III, Medical University of Vienna, Waehringer Guertel 18-20, 1090 Vienna, Austria; 2 Division of Cardiology, Department of Medicine II, Medical University of Vienna, Waehringer Guertel 18-20, 1090 Vienna, Austria; 3 Division of Palliative Medicine, Department of Medicine I, Medical University of Vienna, Waehringer Guertel 18-20, 1090 Vienna, Austria; 4 Department of Medicine I, Medical University of Vienna, Waehringer Guertel 18-20, 1090 Vienna, Austria

**Keywords:** Palliative care, Solid organ tumors, Metastasis, Timely referral, Timing of consultation, Hospitalization, health care expenditures

## Abstract

**Background:**

Cancer is a leading cause of death, often accompanied by significant symptom burden. Palliative care (PC) improves quality of life, clarifies treatment goals, and prevents unnecessary interventions at the end of life. While metastatic disease is considered incurable and routinely leads to PC involvement, the need for PC in patients with non-metastatic cancer is often less clear. This study investigated whether metastatic status affects timing of in-hospital PC consultation and hospitalization length in patients with advanced cancer.

**Methods:**

We retrospectively analyzed hospitalized patients with cancer who received PC consultations between 2016–2022. Outcomes included time from admission to PC consultation, length of hospitalization, in-hospital mortality, and overall survival, analyzed using Kaplan–Meier estimates and Cox regression models.

**Results:**

Among 741 inpatients with cancer, 52.1% had metastatic disease. Patients with non-metastatic cancer received PC involvement later after admission than those with metastatic cancer (median: 9 [95% CI: 7–11] days vs. 7 [95% CI: 6–8] days; *p* = 0.004), and were less likely transferred to the in-hospital PC unit (24.6% vs. 18.0%, *p* = 0.03). Early PC (within 72 h) was numerically less frequent in non-metastatic disease (16.9% vs. 22.5%; *p* = 0.055), and associated with shorter hospital stays (HR: 0.966 [0.961–0.971]; *p* < 0.001), independent of prevalent metastases (HR: 1.14 [0.99–1.33]; *p* = 0.08).

**Conclusions:**

Inpatients with non-metastatic cancer experienced later PC consultation after admission, while overall rates of early PC consultation remained low. Early PC after admission was associated with shorter hospital stays regardless of metastatic status, highlighting the need for early PC during hospitalization across all cancer stages.

**Supplementary Information:**

The online version contains supplementary material available at https://doi.org/10.1007/s00520-026-11196-7.

## Introduction

Cancer is one of the leading causes of death worldwide [1, 2]. Despite advances in targeted therapies and immunotherapies that have improved survival in selected malignancies, metastatic disease remains associated with poor prognosis, substantial symptom burden, and a high risk of medical complications [2, 3]. In this context, palliative care (PC) represents an essential component of comprehensive oncologic treatment, aiming to improve quality of life by addressing physical, psychosocial, and spiritual issues in individuals with advanced illness [4]. Beyond symptom control, PC has also been associated with prolonged survival, and current guidelines recommend its early integration, typically within 8 weeks after diagnosis [5–15]. However, such time-based recommendations may not adequately reflect individual patient needs [15].

Timely integration of PC for patients with advanced cancer has emerged as a refinement of the concept of “early PC”, shifting from time-based referral criteria toward a more individualized approach that considers clinical context and patient needs [15]. In practice, both concepts are largely rooted in the outpatient setting, aiming to integrate PC proactively alongside oncologic care [15].

Despite these recommendations, first contact with PC is still frequently initiated during hospitalizations, often in response to acute clinical deterioration or unmet needs [16]. In this context, inpatient PC consultations may represent a pragmatic opportunity to identify patients with unmet PC needs, although they do not fully reflect the principles of outpatient, needs-based integration.

Previous studies have shown that early PC consultation during hospitalization is associated with shorter hospital stays, lower in-hospital mortality, and improved documentation of end-of-life preferences [17–20]. At the same time, access to in-hospital PC varies across patient groups [16]. Although hospitalized patients with cancer tend to receive PC consultations earlier than those with non-malignant conditions, it remains unclear whether specific disease characteristics like the presence of distant metastases trigger earlier PC involvement in the inpatient setting [16].

Therefore, this study aimed to investigate whether the presence of metastases in patients with cancer is associated with earlier PC consultations during hospitalization. A secondary objective was to examine whether earlier PC referrals are associated with shorter hospital stays and lower inpatient mortality, taking metastatic status into account.

## Methods

This retrospective study analyzed data from patients with solid organ tumors who received their first contact with PC as PC consultation during an index hospitalization at the University Hospital Vienna between 2016 and 2022. The index hospitalization was defined as the first hospital admission during the study period in which a PC consultation occurred. Structured data were extracted from the institutional database, including demographic characteristics, oncologic disease, hospitalization details, and PC consultations. Retrieved oncologic data included tumor category, prevalence of distant metastasis, localization, and number of metastatic sites. Where database entries were incomplete or unclear, patient records were reviewed manually to ensure data accuracy. Patients with any prior contact with PC services, including outpatient consultations, were not included. Each patient was included only once in the analysis. In cases of multiple hospitalizations during the study period, only the index hospitalization with the first PC consultation was considered to avoid intra-individual correlation due to repeated hospitalizations. This study was approved by the institutional ethics committee (EK#: 1333/2019). The primary outcome was the interval from hospital admission to the first PC request. Additional endpoints included hospitalization time; the duration from the first PC request to discharge or in-hospital death, the number of follow-up requests, and transfer to the in-hospital PC unit. Oncologic endpoints included the time from the cancer diagnosis to PC involvement, and overall survival from the time of cancer diagnosis.

### Statistical analysis

Descriptive data are reported as medians with interquartile ranges (IQR). Categorical variables were compared using the Chi^2^ test, while continuous variables were analyzed with the Mann–Whitney U test. Time-to-event outcomes were evaluated between individuals with and without distant metastasis using log-rank tests. Hospitalization time was analyzed according to whether a PC request was made early (within 72 h of admission), as defined previously [18, 21, 22]. Results were displayed as medians with 95% confidence intervals (95% CI) and illustrated with Kaplan–Meier survival plots.

For adjustment, Cox proportional hazards models were applied, incorporating presence of distant metastasis, age at hospitalization, and sex as covariates. The analysis of the hospitalization time was additionally adjusted for time from admission to PC consultation. Results of the Cox proportional hazards models were displayed as hazard ratios (HR) with corresponding 95% CIs. The proportional hazards assumption was verified through graphical inspection using log-minus-log survival plots. Statistical significance was defined as a p-value below 0.05. Analyses were conducted using SPSS (version 23) and R (version 4.4.1).

## Results

### Patient characteristics and differences by metastatic disease status

A total of 741 inpatients with cancer were included (Supplementary Figure S1), of whom 386 (52.1%) had documented distant metastasis at the time of hospitalization (Table 1). The proportion of women was higher in the group with metastatic disease compared with the group with non-metastatic disease (*n* = 219 [56.7%] vs. *n* = 159 [44.8%], *p* = 0.001). Patients with metastatic disease were younger both at cancer diagnosis and at hospital admission, and the distribution of primary tumor sites differed significantly between groups (all *p* < 0.001). Detailed patient and tumor characteristics according to metastatic status are presented in Table 1.

**Table 1:** Patient demographics according to metastatic status

	Non-metastatic disease (*n* = 355)	Metastatic disease (*n* = 386)	*p*-value
Female – no. (%)	159 (44.8%)	219 (56.7%)	0.001^a^
Age at Diagnosis – years	67 (57–74)	60 (50–69)	< 0.001^b^
Age at hospital admission – years	68 (58–77)	64 (54–73)	< 0.001^b^
Age at death – years	69 (59–77)	64 (54–73)	< 0.001^b^
Primary disease – no. (%)			< 0.001^a^
- Lung cancer – no. (%)	68 (19.2%)	60 (15.5%)	
- Gastrointestinal cancer – no. (%)	101 (28.5%)	104 (26.9%)	
- Head and neck cancer – no. (%)	60 (16.9%)	15 (3.9%)	
- Brain tumor – no. (%)	22 (6.2%)	3 (0.8%)	
- Renal/Urothelial cancer – no. (%)	19 (5.4%)	30 (7.8%)	
- Breast and gynecological cancers – no. (%)	46 (13.0%)	103 (26.7%)	
- Prostate cancer – no. (%)	7 (2.0%)	25 (6.5%)	
- Skin cancer – no. (%)	10 (2.8%)	27 (7.0%)	
- Other – no. (%)	22 (6.2%)	19 (4.9%)	
Charlson comorbidity index	5 (5–6)	9 (8–10)	< 0.001^b^
**Modified Charlson comorbidity index (without metastases)**	**5 (5–6)**	**5 (4–6)**	** < 0.001**^**b**^
PC requests during hospitalization – no			0.32 ^b^
1 PC request– no. (%)	322 (90.7%)	351 (90.9%)	
2 PC requests– no. (%)	25 (7.0%)	29 (7.5%)	
≥ 3 PC requests– no. (%)	8 (2.3%)	6 (1.6%)	
**First PC request within 72 h after hospital admission**	**60 (16.9%)**	**87 (22.5%)**	**0.055 **^**a**^
**Transfer to palliative care unit (within the same hospital)**	**64 (18.0%)**	**95 (24.6%)**	**0.03 **^**a**^
Transfer to another health care provider (not specified)	49 (13.8%)	56 (14.5%)	0.78 ^a^
Discharged – no. (%)	118 (33.2%)	140 (36.3%)	0.39 ^a^
In-hospital death – no. (%)	188 (53.0%)	190 (49.2%)	0.31 ^a^
Death after discharge – no. (%)	150 (42.3%)	186 (48.2%)	0.11 ^a^

Metric parameters are displayed by medians and interquartile ranges.

Counts are presented as absolute number and percentage of the respective groups

^a^chi^2^ test, ^b^Mann–Whitney U test

**Fig. 1:**
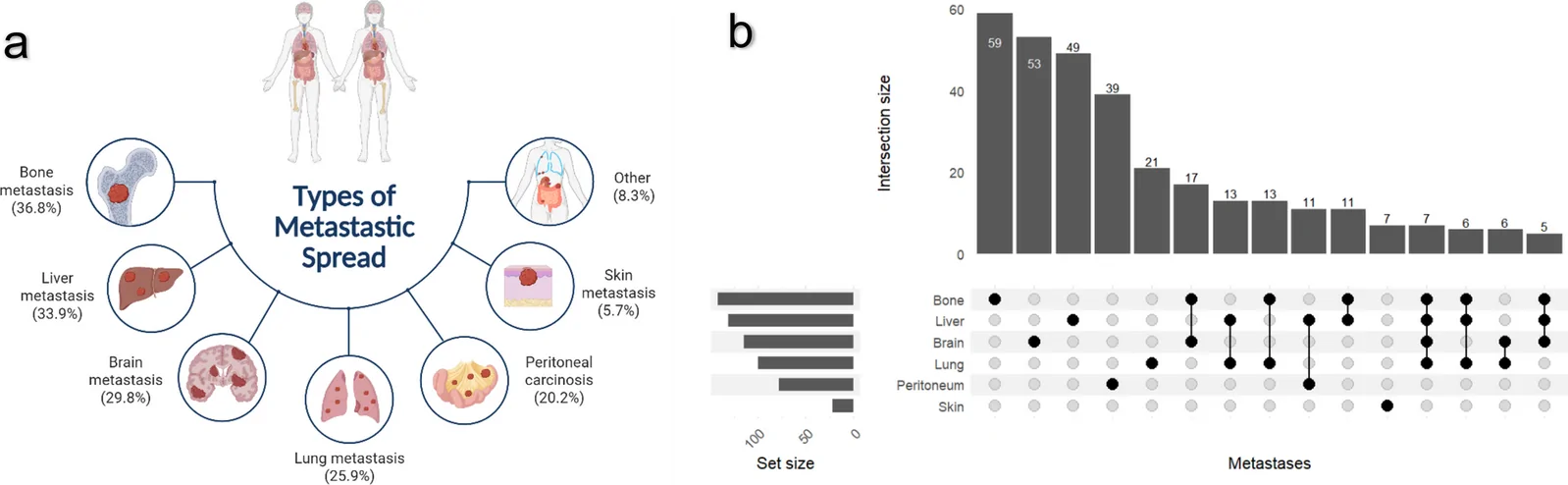
Metastatic distribution in frequently involved organs. Panel **A** depicts the frequency with which individual organs were involved by metastatic disease. Panel **B** presents the combinations of organ involvement using an upset plot. Panel A was created in BioRender. Paschen, C. (2026) https://BioRender.com/ezxphkp

Among patients with metastatic disease, *n* = 231 (59.8%) had metastases affecting a single organ site, *n* = 103 (26.7%) had two metastatic sites, and, *n* = 52 (13.5%) had generalized metastases (≥ 3 sites involved). Figure 1 displays the distribution of metastasis-affected organs.

The unadjusted Charlson Comorbidity Index was higher among individuals with metastatic disease (median: 9 [IQR: 8–10] vs. 5 [IQR: 5–6], *p* < 0.001), reflecting the inclusion of metastatic cancer in the score. After excluding metastases-related scoring (modified Charlson Comorbidity Index), individuals with non-metastatic disease exhibited a higher non-cancer comorbidity burden (median: 5 [IQR: 5–6] vs. 5 [IQR: 4–6], *p* < 0.001).

Patients with metastatic disease had longer intervals between cancer diagnosis and the hospitalization with the first PC involvement (24 months [95% CI: 19–28 months] vs. 10 months [95% CI: 8–12 months]; *p* < 0.001; Table 2) but also to death (27 months [95% CI: 22–31 months] vs. 12 months [95% CI: 9–15 months]; *p* < 0.001).

**Table 2:** Time-to-event analysis according to metastatic status

	Non-metastatic disease (*n* = 355)	Metastatic disease (*n* = 386)	*p*-value
Time from admission to first PC request (days)	9 (7–11)	7 (6–8)	**0.004**
Time from first PC request to discharge (days)	8 (7–9)	8 (6–10)	0.80
Time from first PC request to in-hospital death (days)	7 (6–9)	9 (7–11)	0.41
Length of hospital stay (days)	20 (18–23)	20 (18–22)	0.11
Length of hospital stay (among discharged individuals) (days)	20 (15–26)	18 (15–21)	0.15
Time from admission to in-hospital death (days)	20 (17–24)	22 (19–26)	0.57
Time from discharge to death (days)	30 (24–37)	39 (30–47)	0.68
Survival since diagnosis (months)	12 (9–15)	27 (22–31)	** < 0.001**
Time from diagnosis to first PC request (months)	10 (8–12)	24 (19–28)	** < 0.001**
Time from PC request to death (days)	16 (13–20)	22 (19–26)	0.23

Values are displayed as medians and 95% confidence intervals (CI). Analysis was performed using the log rank test. *PC* Palliative Care

### Timing of in-hospital PC involvement: metastatic vs. non-metastatic disease

**Fig. 2:**
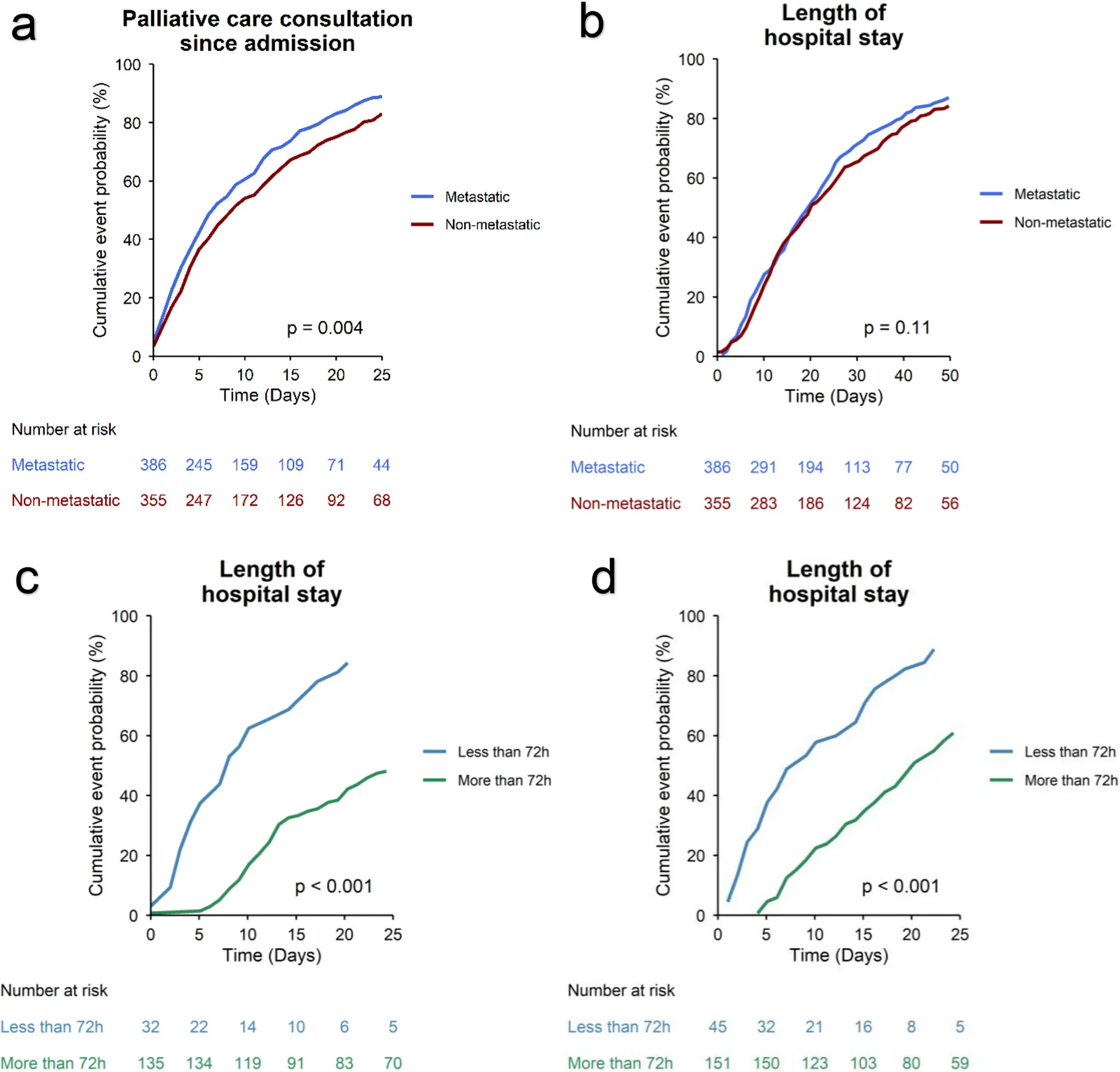
Palliative care (PC) consultation timing and length of hospital stay. Panels A and B show cumulative event probabilities (1-Kaplan–Meier estimate) for time from admission to first in-hospital PC consultation (Panel **A**) and time from admission to discharge, transfer or death (length of hospital stay, Panel **B**), comparing patients with metastatic disease (*n* = 386) and those with non-metastatic cancers (*n* = 355). Panels C and D display length of hospital stay stratified by early PC consultation (within 72 h of admission), shown separately for patients with non-metastatic disease (*n* = 167; Panel **C**) and metastatic disease (*n* = 196; Panel **D**). In Panels C and D, length of hospital stay is presented as cumulative event probability of hospital discharge or transfer, excluding patients who died during hospitalization

Most inpatients in both groups received one PC consultation during their hospital stay (metastatic disease: *n* = 351 [90.9%] vs. non-metastatic disease: *n* = 322 [90.7%]; *p* = 0.32). Individuals with distant metastases received earlier PC involvement following hospital admission, with a median time from admission to first PC consultation of 7 days (95% CI: 6–8 days) compared to 9 days (95% CI: 7–11 days) in individuals with non-metastatic cancers (*p* = 0.004; Fig. 2A). While there were no differences in time from first PC request to discharge (*p* = 0.80), or time from the initial PC consultation to in-hospital death (*p* = 0.41; Table 2), patients with non-metastatic cancer were less likely to be transferred to the in-hospital PC unit (18.0% [*n* = 64] vs. 24.6% [*n* = 95] of patients with metastatic disease, *p* = 0.03).

### Timing of palliative care consultation and length of hospital stay

The overall length of the hospital stay was similar between individuals with and without metastatic disease (median: 20 days [IQR: 18–22 days] vs. 20 days [IQR: 18–23 days], *p* = 0.11; Fig. 2B). Less than a quarter of the cohort received a PC consultation within 72 h of admission, with numerically more frequent PC requests for inpatients with metastases compared to those with non-metastatic solid organ tumors (*n* = 87 [22.5%] vs. *n* = 60 [16.9%], *p* = 0.055).

A PC consultation initiated within 72 h of hospital admission was associated with a significantly shorter hospital stay, regardless of metastatic status (Table 3). Among patients with non-metastatic disease, those who received early PC involvement were significantly younger than those with later consultations (61 years [56–68 years] vs. 68 years [60–76 years]; *p* = 0.006). Sex, primary tumor type, and comorbidity burden did not differ significantly between the groups (Supplementary Table S1). In contrast, there were no age differences between early and late PC consultations among individuals with metastatic cancer (64 years [57–72 years] vs. 65 years [55–74 years]; *p* = 0.84).

**Table 3:** Time-to-event analysis according to metastatic status

		Length of Hospital Stay (days)		Time from first PC request to discharge, transfer or in-hospital death (days)		Time from discharge/transfer to death (days)	
		Median (CI)	*p*-value	Median (CI)	*p*-value	Median (CI)	*p*-value
Non-metastatic disease (*n* = 355)							
Discharge or transfer	< 72 h (*n* = 32)	8 (5–11)	** < 0.001**	7 (5–9)	0.51	21 (14–28)	**0.03**
	> 72 h (*n* = 135)	26 (21–31)		8 (7–9)		31 (22–40)	
In-hospital death	< 72 h (*n* = 28)	9 (4–14)	** < 0.001**	7 (2–12)	0.60		
	> 72 h (*n* = 160)	23 (20–27)		7 (5–9)			
Metastatic disease (*n* = 386)							
Discharge or transfer	< 72 h (*n* = 45)	9 (5–13)	** < 0.001**	8 (4–12)	**0.03**	37 (21–52)	0.72
	> 72 h (*n* = 151)	20 (18–23)		9 (7–11)		40 (31–48)	
In-hospital death	< 72 h (*n* = 42)	7 (5–9)	** < 0.001**	6 (4–8)	0.13		
	> 72 h (*n* = 148)	25 (23–27)		10 (9–12)			

Values are displayed as medians and 95% confidence intervals (CI). Analysis was performed using the log rank test. *PC* Palliative Care

49.2% of individuals (*n* = 190) with prevalent distant metastasis and 53.0% (*n* = 188) of those with non-metastatic disease died in hospital (*p* = 0.31). Accordingly, similar proportions of inpatients with metastatic disease and non-metastatic disease were discharged (*n* = 140 [36.3%] vs. *n* = 118 [33.2%], *p* = 0.39) or transferred to external health care providers (*n* = 56 [14.5%] vs. *n* = 49 [13.8%], *p* = 0.78).

In the subgroup of patients with non-metastatic cancer who were discharged or transferred alive, PC consultation within 72 h of admission was associated with a significantly shorter hospital stay compared to those with later PC involvement (8 days [95% CI: 5–11 days] vs. 26 days [95% CI: 21–31]; *p* < 0.001; Fig. 2C). A similar pattern was observed in individuals with metastatic disease, with shorter hospital stays when PC was involved within 72 h of admission (median: 9 days [95% CI: 5–13 days] vs. 20 days [95% CI: 18–23 days]; *p* < 0.001; Fig. 2D).

### Associations between timing of palliative care and hospital stay

Concordant with the results of the primary analysis, delayed in-hospital PC consultation was significantly associated with longer overall hospital stays (HR: 0.966; 95% CI: 0.961–0.971; *p* < 0.001). Significant triggers for PC consultations were metastatic status (HR: 1.24; 95% CI: 1.07–1.44; *p* = 0.004; Table 4) and female sex (HR: 1.20; 95% CI: 1.04–1.39; *p* = 0.01).

**Table 4:** Cox proportional hazards analysis for inequalities around PC consultation timing

	Time from admission to first PC request (days)		Length of Hospital Stay (days)	
	HR [95% CI]	p-value	HR [95% CI]	*p*-value
Metastatic disease	**1.24 [1.07–1.44]**	**0.004**	1.14 [0.99–1.33]	0.08
Age at hospitalization	1.005 [1.000–1.010]	0.08	**1.010** **[1.004–1.016]**	**0.001**
Female sex	**1.20 [1.04–1.39]**	**0.01**	**0.84 [0.72–0.97]**	**0.02**
Time from admission to first PC request	**-**	**-**	**0.966 [0.961–0.971]**	** < 0.001**

Results are displayed as hazard ratios (HR) and 95% confidence intervals (95% CI). All models were adjusted for the primary diagnosis, age at hospitalization, and patients’ sex. The model length of hospital stays was further adjusted for time from admission to first palliative care (PC) request

Although metastatic status was associated with earlier PC referral, it was not associated with the length of hospital stay (HR: 1.14; 95% CI: 0.99–1.33; *p* = 0.08). Female sex was associated with longer hospital stays (HR: 0.84; 95% CI: 0.72–0.97; *p* = 0.02), while older age was significantly associated with shorter hospital stays (HR: 1.010; 95% CI: 1.004–1.016; *p* = 0.001), but not with timing of PC referral (Table 4).

## Discussion

Taken together, our findings show that individuals with non-metastatic cancer received PC consultations later than those with metastatic disease and were less frequently transferred to the specialized inpatient PC unit. Fewer than 20% of hospitalized patients received an early PC consultation within the first 72 h after admission, although this proportion was notably higher among individuals with metastatic cancer. Both in-hospital and overall mortality, and the time from PC consultation to death were similar between individuals with and without metastases. Early PC involvement was independently associated with shorter hospital stays in both groups. Among patients with non-metastatic cancer who were discharged alive, those receiving early PC consultations were significantly younger than those with later consultations, whereas no age differences were observed among discharged patients with metastatic disease. By analyzing PC consultation patterns according to metastatic status, a key indicator of incurable disease in cancer [2], our findings extend previous work on this topic [18–21] and underscore the relevance of early inpatient PC involvement irrespective of disease stage.

These findings are consistent with previous studies demonstrating that earlier in-hospital PC involvement is associated with shorter hospital stays [18, 21, 22]. However, prior analyses often investigated heterogeneous inpatient populations in which patients with cancer accounted for 11% to 47.8% of the population, limiting conclusions for this subgroup [18–22]. Moreover, consultation timing may vary substantially by primary diagnosis, thereby confounding interpretation for patients with cancer [16, 23]. By focusing exclusively on individuals with solid organ tumors and differentiating between non-metastatic and metastatic disease, our study provides a more granular perspective. In this context, a subgroup analysis by Davis et al. did not observe an association between early PC consultation and shorter hospital stays among inpatients with cancer [18]. In contrast, we observed an association between earlier PC consultation and shorter hospital stays in both patients with metastatic and non-metastatic disease.

The observed delay in PC consultations among individuals with non-metastatic disease raises an important clinical question: why are these individuals referred more hesitantly than those with metastatic disease? While metastatic disease is widely regarded as incurable, non-metastatic disease, even when advanced, often leaves room for therapeutic ambiguity. Curative treatment may still be possible, yet decreased functional status or acute deterioration can temporarily preclude disease-directed therapy. Conversely, recognizing when locally advanced disease has become incurable is crucial for PC integration. Uncertainty regarding prognosis and treatment intent may delay referral, postponing symptom management, psychosocial support, and advance care planning. Notably, although overall comorbidity burden appeared higher among patients with metastatic disease, this difference was largely driven by metastasis-related weighting within the Charlson Comorbidity Index, whereas patients with non-metastatic disease exhibited a higher comorbidity burden when these points were excluded, underscoring their substantial clinical complexity despite the absence of distant metastases [24]. This is further supported by the comparable mortality and from PC consultation to death observed in both groups, suggesting that delayed PC involvement in non-metastatic cancer may not be explained by a more favorable prognosis and may point toward potential underutilization of PC in this population.

In addition to metastatic status, female sex was independently associated with earlier PC consultation. Although the underlying mechanisms cannot be determined from our data, possible explanations include sex-related differences in symptom expression, help-seeking behavior, and communication with healthcare providers [25–27]. Previous studies suggest that women may be more likely to report pain and emotional distress, potentially facilitating discussions about supportive care, whereas men may adopt more treatment-focused or stoic coping strategies, which could delay the integration of needs-based PC [25–27]. Differences in clinicians’ perception of PC needs according to sex may also contribute to the observed association [28, 29].

Importantly, these findings should be interpreted in the context of the broader conceptual shift from early to timely PC. Early PC typically refers to predefined, often time-based integration early after diagnosis, usually in the outpatient setting, whereas timely PC emphasizes needs-based, proactive integration according to individual patient needs [15]. Professional guidelines recommend integration of PC early after diagnosis, ideally in the outpatient setting [15, 30, 31], although not all patients exhibit PC needs at that time [15]. Even stepwise approaches to outpatient PC, initiated in response to changes in oncologic trajectories (disease progression, change of treatment regimens or following hospitalizations) have been shown to provide outcomes comparable to continuous PC with regular visits [32]. In contrast, our analysis captures PC delivery in hospitalized patients and therefore reflects a more reactive stage of care, in which PC consultation timing is primarily driven by acute clinical events rather than systematically assessed needs [33].

After admission, patients with metastatic disease may be more readily recognized as having an incurable condition, prompting earlier discussions about goals of care. However, metastatic disease is heterogeneous, and some patients may live for years with relatively stable disease [2, 3]. Conversely, patients with non-metastatic tumors may experience substantial symptom burden despite the absence of metastases. These differences likely contribute to variability in referral patterns and highlight that metastatic status alone is an imperfect proxy for cancer patients’ PC needs.

Hospitalization itself, though not typically considered a formal oncologic milestone, may represent a critical moment for PC integration. Many patients with cancer are hospitalized during the last three months of life, and acute deterioration often signals a turning point in the disease trajectory [33–36]. In this context, any hospital admission for patients with cancer, whether non-metastatic or metastatic, should prompt clinicians to reassess PC needs, regardless of metastatic status. This is particularly relevant given that most patients with cancer prefer to spend their final phase of life outside the hospital, highlighting the importance of early PC involvement to facilitate appropriate discharge planning, including transitions to home-based care or hospice services [21, 33, 37]. However, providing high-quality end-of-life care at home requires specialized PC teams capable of home visits and relatives who are willing and able to provide support [38]. When these conditions are met, patients’ end-of-life preferences can be more effectively honored.

Beyond the patient-centered perspective, PC also has economic implications. The association between earlier PC consultation and shorter length of stay in our study supports its role in healthcare resource utilization. A substantial proportion of lifetime healthcare expenditures occur during the final months of life [39, 40], and early PC involvement has been associated with reduced resource use [41], potentially through improved alignment of care with patient preferences and avoidance of non-beneficial interventions [9, 17, 37]. In the inpatient setting, PC consultations initiated within two days of admission have been linked to greater cost reductions of approximately 24% compared with no PC involvement [42]. Our findings are consistent with this perspective, as earlier PC consultation was associated with shorter hospital stays.

This study has several limitations. The study design was based on administrative data and therefore did not capture detailed clinical information. In particular, detailed information on local tumor extent was unavailable, meaning that the non-metastatic group may have included both patients with early-stage disease and those with locally advanced non-metastatic cancer. Likewise, we could not evaluate whether known or newly diagnosed metastases triggered PC involvement, nor could we assess treatment intent, symptom burden, psychosocial distress, patient help-seeking behavior, clinician referral practices or acute clinical deterioration. Consequently, residual confounding by indication cannot be excluded, and the observed association between earlier PC involvement and shorter hospital stay should not be interpreted as evidence of a causal effect. Importantly, we could not evaluate whether PC consultations were aligned with patient needs, which represents a key component of the concept of timely, needs-based PC. Consequently, our findings reflect patterns of PC utilization rather than appropriateness of referral.

Despite these limitations, our study provides insight into real-world PC delivery in hospitalized patients with cancer. The observed differences in PC consultation timing and transfer to the PC unit between non-metastatic and metastatic disease highlight potential gaps in referral practices and suggest that reliance on disease stage alone may delay PC involvement. Future prospective studies are needed to better define clinical triggers for PC consultation, rather than oncologic milestones alone, to better achieve needs-based PC in outpatient and inpatient settings.

## Conclusions

In conclusion, the present study provides valuable insights indicating that individuals with non-metastatic cancer are less likely to receive timely PC involvement after hospital admission compared with those with metastatic solid organ tumors. However, overall early PC consultation rates remained low, even though timely PC engagement was associated with shorter hospital stays, independent of metastatic status. These results suggest that hospitalization should prompt the early involvement of PC providers across all cancer stages to improve patient-centered outcomes and optimize the use of healthcare resources.

## Supplementary Information

Below is the link to the electronic supplementary material.

Supplementary file1 (DOCX 54 KB)

Supplementary file2 (DOCX 35.6 KB)

## Data Availability

The datasets used and/or analyzed during the current study are available from the corresponding author on reasonable request.

## Declarations

### Declaration of generative artificial intelligence use

The authors used ChatGPT v5.3 for English language editing. The authors reviewed and edited the content as needed and take full responsibility for the content of the publication.

## Abbreviations

*CI*: Confidence interval
*HR*: Hazard ratio
*IQR*: Interquartile range
*PC*: Palliative care
*SD*: Standard deviation
