# Supplementary material for: Missed opportunities: delayed in-hospital palliative care consultations in patients with non-metastatic cancers
Source: Support Care Cancer. 2026 Sep 9;34(10):949. doi: 10.1007/s00520-026-11196-7 (PMC13558332; doi:10.1007/s00520-026-11196-7)
Supplement: Supplementary file 1 [file 520_2026_11196_MOESM1_ESM.docx]

**Missed Opportunities: Delayed In-Hospital Palliative Care Consultations in Patients with Non-Metastatic Cancers**

**Supplementary Material**

| Supplementary Table S1: Characteristics of non-metastatic patients discharged alive,  stratified by timing of the first PC consultation after admission (n=167) | | | |
| --- | --- | --- | --- |
|  | First PC consultation  <72 hours after  admission (n=32) | First PC consultation  >72 hours after  admission (n=135) | p-value |
| Female – no. (%) | 15 (46.9%) | 56 (41.5%) | 0.58 ^a^ |
| **Age at Diagnosis – years** | **60 (52-67)** | **67 (58-74)** | **0.003 ^b^** |
| **Age at Palliative Care Consultation – years** | **61 (56-68)** | **68 (60-76)** | **0.006 ^b^** |
| Primary disease – no. (%) |  |  | 0.46 ^a^ |
| - Lung cancer – no. (%) | 4 (12.5%) | 25 (18.5%) |  |
| - Gastrointestinal cancer – no. (%) | 8 (25.0%) | 44 (32.6%) |  |
| - Head and neck cancer – no. (%) | 8 (25.0%) | 24 (17.8%) |  |
| - Brain tumor – no. (%) | 2 (6.3%) | 13 (9.6%) |  |
| - Renal/Urothelial cancer – no. (%) | 2 (6.3%) | 4 (3.0%) |  |
| - Breast and gynecological cancers – no. (%) | 4 (12.5%) | 15 (11.1%) |  |
| - Prostate cancer – no. (%) | 0 (-) | 2 (1.5%) |  |
| - Skin cancer – no. (%) | 0 (-) | 3 (2.2%) |  |
| - Other – no. (%) | 4 (12.5%) | 5 (3.7%) |  |
| Charlson comorbidity index | 5 (4-6) | 5 (5-6) | 0.20 ^b^ |
| Legend: Metric parameters are displayed by medians and interquartile ranges.  Counts are presented as absolute number and percentage of the respective groups. a chi² test, b Mann-Whitney U test | | | |

Supplementary Figure S1: Study flow chart.

**Hospitalized individuals with cancer (n = 741)**

**Hospitalized individuals at the Vienna General Hospital who received a palliative care request between 03/2016 and 08/2022 (n = 918)**

**Excluded due to:**

- **Other primary diagnosis than a solid organ tumor (n = 122)**
- **Insufficient documentation on oncologic disease status (n = 55)**

**Non-metastatic Disease
(n = 355)**

**Metastatic
Disease
(n = 386)**
